# 2,2′-[(4-But­oxy­phen­yl)methyl­ene]bis­(3-hy­droxy-5,5-di­methyl­cyclo­hex-2-en-1-one)

**DOI:** 10.1107/S2414314625001804

**Published:** 2025-02-28

**Authors:** N. Suresh Babu, S. Anbu Chudar Azhagan, B. Loganathan, V. Sughanya, J. Ayyappan

**Affiliations:** a Department of Chemistry, Government College of Engineering, Tirunelveli-627 007, Tamilnadu, India; b Department of Physics, Government College of Engineering, Tirunelveli-627 007, Tamilnadu, India; c Department of Chemistry (Science and Humanities), Dr. N. G. P. Institute of Technology, Coimbatore-641 048, Tamil Nadu, India; d Department of Chemistry, Periyar Government Arts College, Cuddalore-607 001., Tamil Nadu, India; e Department of Physics, Government College of Engineering, Salem-636 011, Tamilnadu, India; Katholieke Universiteit Leuven, Belgium

**Keywords:** crystal structure, 4-but­oxy­benzaldehyde, dimedone, xanthene, C—H⋯π (ring) inter­action, hydrogen bond

## Abstract

In the title compound, C_27_H_36_O_5_, the dihedral angles between the planes of the benzene ring and the cyclo­hexenone rings are 60.87 (10) and 65.04 (10)°, while the dihedral angle between the mean planes of the two cyclo­hexenone rings is 39.33 (10)°. Each cyclo­hexenone ring has a carbon atom bonded to two methyl groups, which acts as the flap atom, resulting in an envelope conformation. The opposite orientation of the hy­droxy and carbonyl oxygen atoms allows for the formation of two intra­molecular O—H⋯O hydrogen bonds and C—H⋯π (ring) inter­actions also help to establish the molecular conformation.

## Abstract 2

In the title compound, C_27_H_36_O_5_, both the cyclo­hexenone rings adopt envelope conformations.

## Graphical abstract

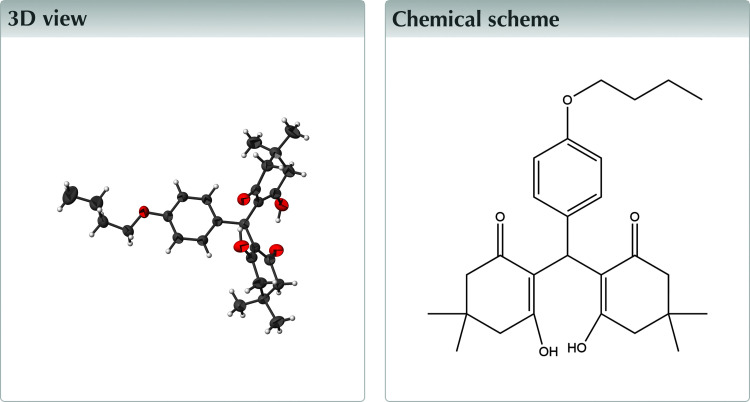

## Structure description

Xanthene derivatives possess biological properties such as anti­viral, anti­bacterial (Dimmock *et al.*, 1988 ▸) and anti-inflammatory (Dimmock *et al.*, 1988 ▸; Cottam *et al.*, 1996 ▸) activities and are therefore used in medicine. Xanthene is present in organic compounds that are widely used as synthetic dyes (Hilderbrand *et al.*, 2007 ▸), in laser technologies (Pohlers *et al.*, 1997 ▸) and in fluorescent materials used for visualization of biomolecules (Knight & Stephens, 1989 ▸; Khan & Sekar, 2023 ▸; Majumdar *et al.*, 2022 ▸; Lakhrissi *et al.*, 2022 ▸).

**Figure 1:**
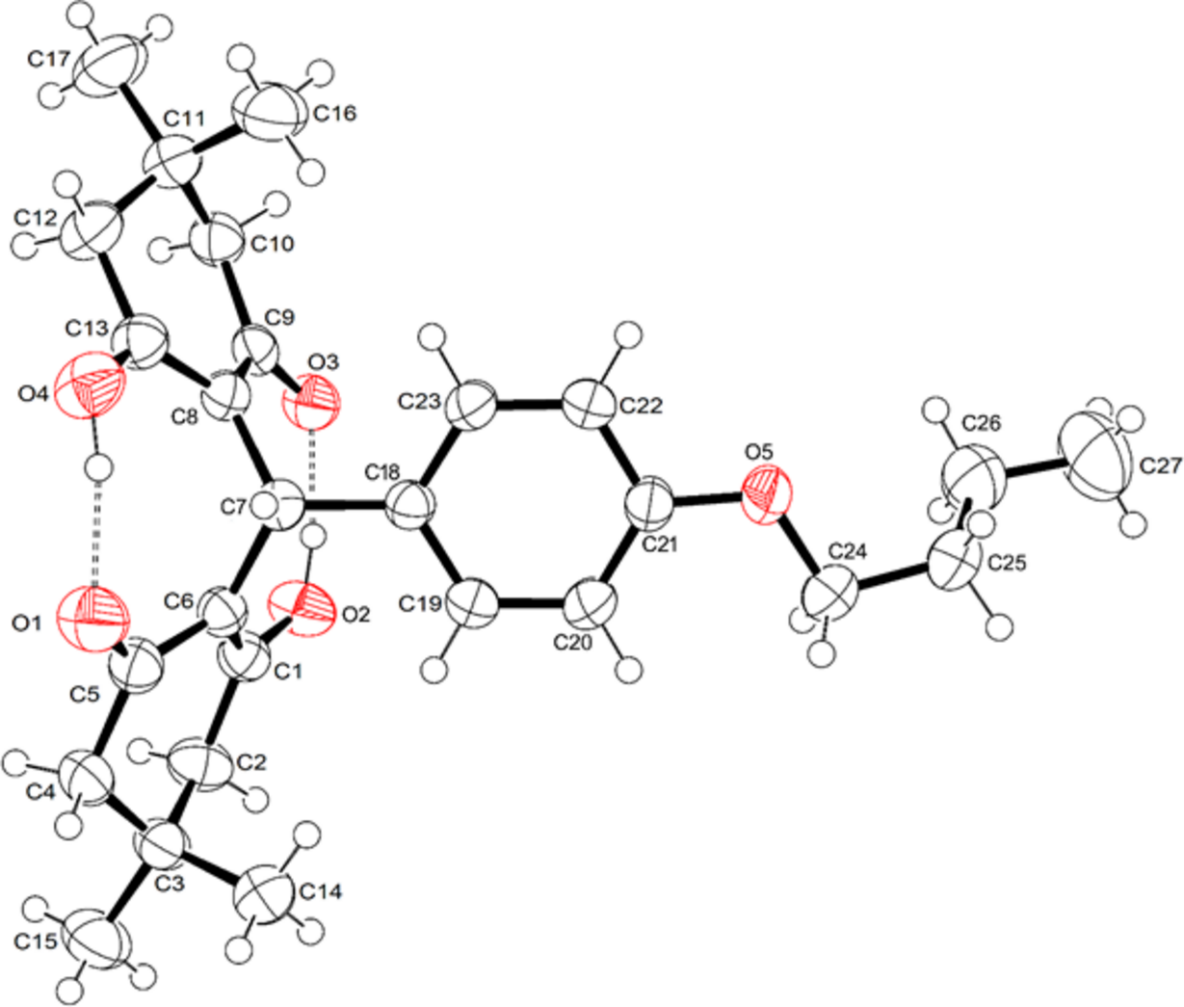
The mol­ecular structure of the title compound showing the atom-numbering scheme and intra­molecular O—H⋯O hydrogen bonds as dashed lines. Displacement ellipsoids are drawn at the 50% probability level.

In the title compound (Fig. 1 ▸), the bond lengths (Allen *et al.*, 1987 ▸) and angles are close to those reported for similar compounds (for examples, see: Sureshbabu & Sughanya, 2012 ▸, 2013 ▸; Sughanya & Sureshbabu, 2012 ▸; Khalilov *et al.*, 2023 ▸; Steiger *et al.*, 2020 ▸). In the cyclo­hexenone ring, C1—C6 and C8—C13 are double bonds, as indicated by the bond lengths [1.380 (2) and 1.374 (2) Å, respectively]. The observed carbonyl bond lengths [C5—O1 = 1.270 (2) and C9—O3 = 1.268 (2) Å] are also normal. Cyclo­hexenone rings *A* (C1–C6) and *B* (C8–C13) are not planar with total puckering amplitudes *Q*(T) of 0.466 (2) Å (for *A*) and 0.472 (2) Å (for *B*). Atoms C3 and C11 act as the flap atoms in the envelope conformations of cyclo­hexenone rings *A* and *B*, deviating from the mean planes of the rings by 0.325 (2) and 0.327 (2) Å, respectively. The observed conformations can be described by the puckering parameters (Cremer & Pople, 1975 ▸), which yield values of φ = 122.3 (3)° and θ = 63.5 (2)° for ring *A* and φ = 186.8 (3)° and θ = 63.5 (2)° for ring *B*. Ring *A* and *B* make dihedral angles of 60.87 (10) and 65.04 (10)°, respectively, with the benzene ring. The dihedral angle between the mean planes of the cyclo­hexenone rings is 39.33 (10)°.

**Figure 2:**
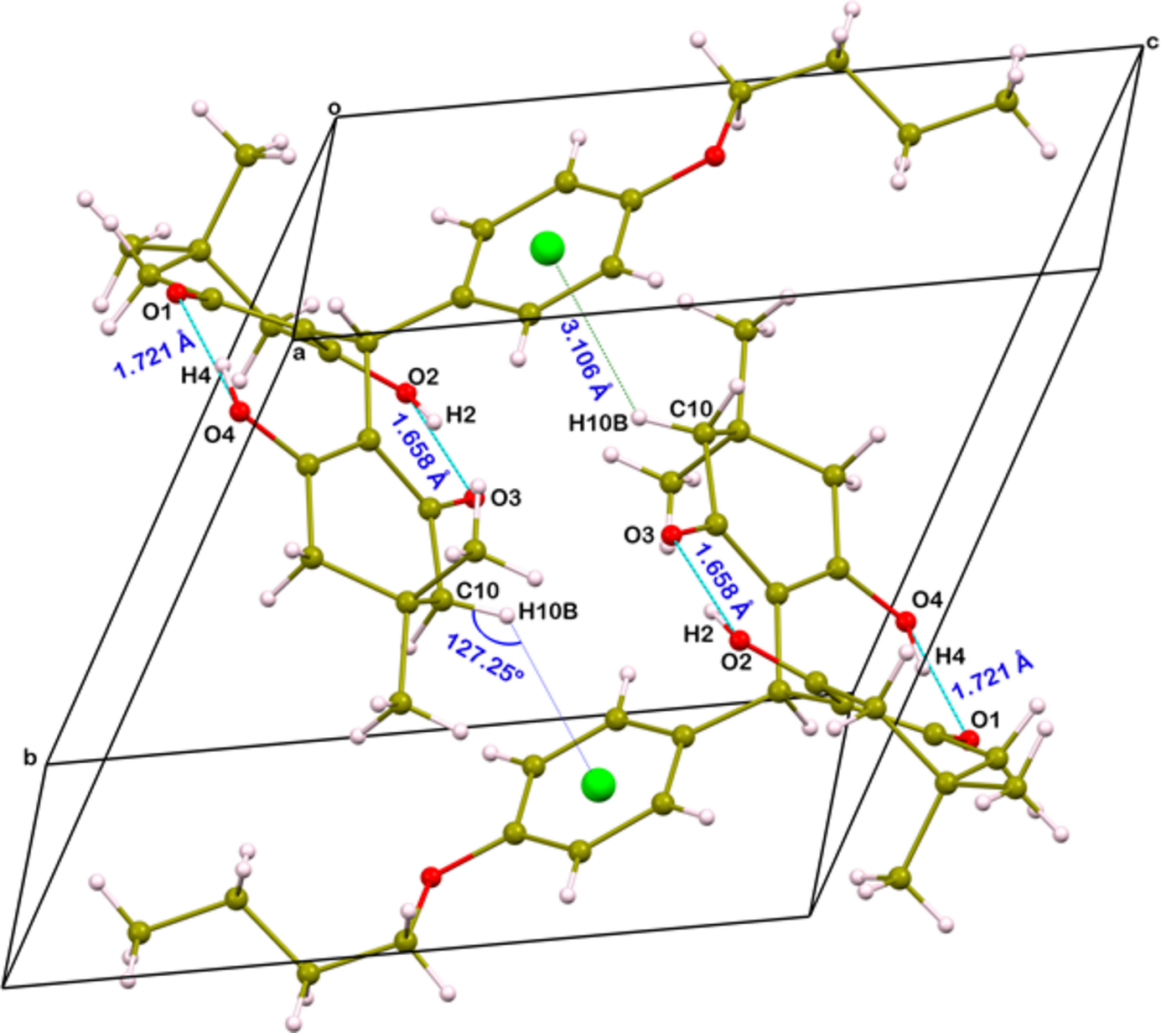
A view of the packing in the crystal structure, showing the O—H⋯O hydrogen bonds and C—H⋯π inter­actions as dashed lines.

The structure features two intra­molecular O—H⋯O hydrogen bonds (Fig. 1 ▸, Table 1 ▸). The relatively short H⋯*A* distances and the near-linear *D*—H⋯*A* angles suggest that these are strong hydrogen bonds. An inter­molecular C10—H10*B*⋯π(C18–C22) inter­action is also observed (Fig. 2 ▸, Table 1 ▸).

## Synthesis and crystallization

The title compound was prepared in a single stage as previously described (Horning & Horning, 1946 ▸; Kaupp *et al.* 2003 ▸). A mixture consisting of 4-but­oxy­benzaldehyde (0.712 g, 4 mmol), 5,5-di­methyl­cyclo­hexane-1,3-dione (1.12 g, 8 mmol) and 15 ml of ethanol was heated at 343 K for approximately 5 minutes. The reaction mixture was allowed to cool to room temperature and the resulting title compound was filtered and dried. Colourless crystal were obtained by crystallization from ethanol solution at room temperature, m.p. 401 K, yield 1.65 g (3.75 mmol, 94%).

## Refinement

Crystal data, data collection, and refinement details are summarized in Table 2 ▸.

## Supplementary Material

Crystal structure: contains datablock(s) I. DOI: 10.1107/S2414314625001804/vm4064sup1.cif

Structure factors: contains datablock(s) I. DOI: 10.1107/S2414314625001804/vm4064Isup2.hkl

Supporting information file. DOI: 10.1107/S2414314625001804/vm4064Isup3.cml

CCDC reference: 2421922

Additional supporting information:  crystallographic information; 3D view; checkCIF report

## Figures and Tables

**Table 1:** Hydrogen-bond geometry (Å, °) *Cg*1 is the centroid of the C18–C22 benzene ring.

*D*—H⋯*A*	*D*—H	H⋯*A*	*D*⋯*A*	*D*—H⋯*A*
O2—H2⋯O3	0.92 (2)	1.66 (2)	2.566 (2)	166 (3)
O4—H4⋯O1	0.94 (3)	1.72 (3)	2.655 (2)	171 (3)
C10—H10*B*⋯*Cg*1^i^	0.97	3.11	3.773 (2)	127

Symmetry code: (i) 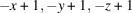.

**Table 2:** Experimental details

Crystal data	
Chemical formula	C_27_H_36_O_5_
*M*_r_	440.56
Crystal system, space group	Triclinic, *P* 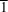
Temperature (K)	296
*a*, *b*, *c* (Å)	10.3372 (14), 11.3286 (15), 12.4559 (16)
α, β, γ (°)	105.428 (7), 114.185 (7), 97.344 (8)
*V* (Å^3^)	1235.3 (3)
*Z*	2
Radiation type	Mo *K*α
μ (mm^−1^)	0.08
Crystal size (mm)	0.20 × 0.15 × 0.12

Data collection	
Diffractometer	Bruker kappa APEXII
Absorption correction	Multi-scan (*SADABS*; Krause *et al.*, 2015 ▸ )
*T*_min_, *T*_max_	0.904, 0.983
No. of measured, independent and observed [*I* > 2σ(*I*)] reflections	29655, 6476, 3194
*R*_int_	0.084
(sin θ/λ)_max_ (Å^−1^)	0.680

Refinement	
*R*[*F*^2^ > 2σ(*F*^2^)], *wR*(*F*^2^), *S*	0.052, 0.145, 1.02
No. of reflections	6476
No. of parameters	303
No. of restraints	2
H-atom treatment	H atoms treated by a mixture of independent and constrained refinement
Δρ_max_, Δρ_min_ (e Å^−3^)	0.19, −0.15

Computer programs: *APEX3* and *SAINT* (Bruker, 2016 ▸), *SHELXT* (Sheldrick, 2015*a* ▸), *SHELXL* (Sheldrick, 2015*b* ▸), *ORTEP-3 for Windows* and *WinGX* (Farrugia, 2012 ▸), *Mercury* (Macrae *et al.*, 2020 ▸), *publCIF* (Westrip, 2010 ▸) and *PLATON* (Spek, 2020 ▸).

## full crystallographic data

### 2,2'-[(4-Butoxyphenyl)methylene]bis(3-hydroxy-5,5-dimethylcyclohex-2-en-1-one) Crystal data

**Table:**

C_27_H_36_O_5_	*F*(000) = 476
*M**_r_* = 440.56	*D*_x_ = 1.184 Mg m^−^^3^
Triclinic, *P*1	Melting point: 401 K
*a* = 10.3372 (14) Å	Mo *K*α radiation, λ = 0.71073 Å
*b* = 11.3286 (15) Å	Cell parameters from 4681 reflections
*c* = 12.4559 (16) Å	θ = 2.2–23.3°
α = 105.428 (7)°	µ = 0.08 mm^−^^1^
β = 114.185 (7)°	*T* = 296 K
γ = 97.344 (8)°	BLOCK, colourless
*V* = 1235.3 (3) Å^3^	0.20 × 0.15 × 0.12 mm
*Z* = 2	

### 2,2'-[(4-Butoxyphenyl)methylene]bis(3-hydroxy-5,5-dimethylcyclohex-2-en-1-one) Data collection

**Table:**

Bruker kappa APEXII diffractometer	6476 independent reflections
Radiation source: fine-focus sealed tube	3194 reflections with *I* > 2σ(*I*)
Graphite monochromator	*R*_int_ = 0.084
ω and φ scan	θ_max_ = 28.9°, θ_min_ = 1.9°
Absorption correction: multi-scan (*SADABS*; Krause *et al.*, 2015 )	*h* = −14→14
*T*_min_ = 0.904, *T*_max_ = 0.983	*k* = −15→15
29655 measured reflections	*l* = −16→16

### 2,2'-[(4-Butoxyphenyl)methylene]bis(3-hydroxy-5,5-dimethylcyclohex-2-en-1-one) Refinement

**Table:**

Refinement on *F*^2^	Hydrogen site location: mixed
Least-squares matrix: full	H atoms treated by a mixture of independent and constrained refinement
*R*[*F*^2^ > 2σ(*F*^2^)] = 0.052	*w* = 1/[σ^2^(*F*_o_^2^) + (0.0478*P*)^2^ + 0.1871*P*] where *P* = (*F*_o_^2^ + 2*F*_c_^2^)/3
*wR*(*F*^2^) = 0.145	(Δ/σ)_max_ < 0.001
*S* = 1.01	Δρ_max_ = 0.19 e Å^−^^3^
6476 reflections	Δρ_min_ = −0.15 e Å^−^^3^
303 parameters	Extinction correction: SHELXL (Sheldrick, 2015b), Fc^*^=kFc[1+0.001xFc^2^λ^3^/sin(2θ)]^-1/4^
2 restraints	Extinction coefficient: 0.109 (4)

### 2,2'-[(4-Butoxyphenyl)methylene]bis(3-hydroxy-5,5-dimethylcyclohex-2-en-1-one) Special details

**Table:**

Geometry. All esds (except the esd in the dihedral angle between two l.s. planes) are estimated using the full covariance matrix. The cell esds are taken into account individually in the estimation of esds in distances, angles and torsion angles; correlations between esds in cell parameters are only used when they are defined by crystal symmetry. An approximate (isotropic) treatment of cell esds is used for estimating esds involving l.s. planes.
Refinement. All hydrogen atoms were identified from difference in electron density peaks and subsequently treated as riding atoms with d(Csp2 —H) = 0.93 Å, d(Cmethyl—H) = 0.96 Å, d(Cmethylene—H) = 0.97 Å, d(Cmethine—H) = 0.98 Å, d(O—H) = 0.82 Å, and *U*_iso_(H) = *xU*_eq_(C,*O*), where *x* = 1.5 for methyl H and 1.2 for all other H atoms.

### 2,2'-[(4-Butoxyphenyl)methylene]bis(3-hydroxy-5,5-dimethylcyclohex-2-en-1-one) Fractional atomic coordinates and isotropic or equivalent isotropic displacement parameters (Å^2^)

**Table:**

	*x*	*y*	*z*	*U*_iso_*/*U*_eq_	
C1	0.9540 (2)	0.64151 (17)	0.87672 (17)	0.0410 (4)	
C2	1.1067 (2)	0.63643 (19)	0.95588 (18)	0.0522 (5)	
H2A	1.163770	0.643254	0.911360	0.063*	
H2B	1.101053	0.553939	0.965337	0.063*	
C3	1.1885 (2)	0.73860 (18)	1.08581 (17)	0.0474 (5)	
C4	1.0844 (2)	0.74257 (19)	1.14393 (17)	0.0497 (5)	
H4A	1.074848	0.667597	1.166673	0.060*	
H4B	1.128142	0.816283	1.220730	0.060*	
C5	0.9334 (2)	0.74828 (18)	1.06066 (17)	0.0442 (5)	
C6	0.87642 (19)	0.70583 (16)	0.92811 (16)	0.0367 (4)	
C7	0.73019 (18)	0.72894 (16)	0.85046 (15)	0.0361 (4)	
H7	0.719136	0.795891	0.911993	0.043*	
C8	0.59514 (19)	0.61938 (17)	0.80092 (16)	0.0372 (4)	
C9	0.5451 (2)	0.51703 (17)	0.68598 (16)	0.0390 (4)	
C10	0.4007 (2)	0.42026 (19)	0.62870 (18)	0.0485 (5)	
H10A	0.419450	0.342368	0.642254	0.058*	
H10B	0.353068	0.401903	0.538652	0.058*	
C11	0.2941 (2)	0.45827 (19)	0.67898 (19)	0.0521 (5)	
C12	0.3821 (2)	0.5133 (2)	0.82193 (19)	0.0552 (6)	
H12A	0.320767	0.549349	0.855828	0.066*	
H12B	0.405858	0.444667	0.852679	0.066*	
C13	0.5216 (2)	0.61327 (18)	0.87035 (18)	0.0444 (5)	
C14	1.2354 (2)	0.86802 (19)	1.0768 (2)	0.0582 (6)	
H14A	1.296929	0.862935	1.036209	0.087*	
H14B	1.288990	0.931057	1.159736	0.087*	
H14C	1.149520	0.891156	1.028790	0.087*	
C15	1.3263 (2)	0.7069 (2)	1.1670 (2)	0.0706 (7)	
H15A	1.299016	0.625870	1.173580	0.106*	
H15B	1.375905	0.771208	1.249396	0.106*	
H15C	1.390873	0.703723	1.129299	0.106*	
C16	0.2257 (2)	0.5560 (2)	0.6272 (2)	0.0746 (7)	
H16A	0.302038	0.631312	0.653908	0.112*	
H16B	0.157509	0.577284	0.658149	0.112*	
H16C	0.174873	0.520770	0.536970	0.112*	
C17	0.1702 (3)	0.3414 (2)	0.6390 (2)	0.0774 (7)	
H17A	0.104120	0.365868	0.671439	0.116*	
H17B	0.211193	0.279550	0.671525	0.116*	
H17C	0.117397	0.305452	0.548877	0.116*	
C18	0.72888 (19)	0.78827 (16)	0.75292 (15)	0.0361 (4)	
C19	0.8561 (2)	0.84862 (18)	0.75624 (18)	0.0461 (5)	
H19	0.946390	0.844951	0.813861	0.055*	
C20	0.8535 (2)	0.91443 (19)	0.67650 (18)	0.0486 (5)	
H20	0.940970	0.952848	0.680025	0.058*	
C21	0.7207 (2)	0.92277 (17)	0.59181 (17)	0.0415 (4)	
C22	0.5921 (2)	0.86339 (18)	0.58710 (17)	0.0447 (5)	
H22	0.501922	0.867947	0.530217	0.054*	
C23	0.5972 (2)	0.79769 (17)	0.66614 (17)	0.0427 (5)	
H23	0.509472	0.758152	0.661380	0.051*	
C24	0.8338 (2)	1.0409 (2)	0.50532 (19)	0.0535 (5)	
H24A	0.882600	0.975976	0.488203	0.064*	
H24B	0.901890	1.107137	0.585082	0.064*	
C25	0.7873 (2)	1.0956 (2)	0.40203 (19)	0.0562 (6)	
H25A	0.874610	1.143573	0.405629	0.067*	
H25B	0.730690	1.154325	0.416725	0.067*	
C26	0.6965 (3)	0.9969 (3)	0.2723 (2)	0.0763 (7)	
H26A	0.748689	0.933122	0.260941	0.092*	
H26B	0.604385	0.955028	0.265916	0.092*	
C27	0.6634 (4)	1.0494 (3)	0.1690 (2)	0.1080 (11)	
H27A	0.605640	1.108246	0.175882	0.162*	
H27B	0.609445	0.981267	0.089307	0.162*	
H27C	0.753938	1.092567	0.175443	0.162*	
O1	0.85854 (16)	0.78933 (15)	1.11437 (12)	0.0619 (4)	
O2	0.90380 (16)	0.58030 (14)	0.75734 (12)	0.0548 (4)	
O3	0.62230 (14)	0.50085 (12)	0.62903 (11)	0.0487 (4)	
O4	0.56851 (17)	0.69215 (15)	0.98548 (13)	0.0604 (4)	
O5	0.70516 (14)	0.98724 (14)	0.51060 (13)	0.0573 (4)	
H2	0.803 (2)	0.565 (3)	0.712 (2)	0.120 (11)*	
H4	0.670 (2)	0.733 (3)	1.027 (3)	0.167 (15)*	

### 2,2'-[(4-Butoxyphenyl)methylene]bis(3-hydroxy-5,5-dimethylcyclohex-2-en-1-one) Atomic displacement parameters (Å^2^)

**Table:**

	*U*^11^	*U*^22^	*U*^33^	*U*^12^	*U*^13^	*U*^23^
C1	0.0403 (10)	0.0388 (10)	0.0392 (11)	0.0100 (9)	0.0156 (9)	0.0119 (9)
C2	0.0432 (11)	0.0483 (12)	0.0561 (13)	0.0174 (10)	0.0167 (10)	0.0131 (10)
C3	0.0404 (11)	0.0472 (12)	0.0458 (11)	0.0103 (9)	0.0112 (9)	0.0185 (9)
C4	0.0477 (12)	0.0509 (12)	0.0389 (11)	0.0065 (10)	0.0108 (9)	0.0167 (9)
C5	0.0458 (11)	0.0427 (11)	0.0396 (11)	0.0077 (9)	0.0179 (9)	0.0130 (9)
C6	0.0368 (10)	0.0368 (10)	0.0360 (10)	0.0098 (8)	0.0166 (8)	0.0128 (8)
C7	0.0376 (10)	0.0362 (10)	0.0361 (9)	0.0113 (8)	0.0186 (8)	0.0118 (8)
C8	0.0364 (10)	0.0402 (10)	0.0375 (10)	0.0114 (8)	0.0179 (8)	0.0157 (8)
C9	0.0399 (10)	0.0424 (11)	0.0387 (10)	0.0151 (9)	0.0169 (9)	0.0203 (9)
C10	0.0467 (12)	0.0465 (11)	0.0432 (11)	0.0066 (9)	0.0152 (9)	0.0141 (9)
C11	0.0420 (11)	0.0540 (13)	0.0588 (13)	0.0075 (10)	0.0222 (10)	0.0224 (11)
C12	0.0503 (12)	0.0594 (13)	0.0610 (13)	0.0085 (11)	0.0335 (11)	0.0192 (11)
C13	0.0427 (11)	0.0465 (11)	0.0455 (11)	0.0123 (9)	0.0224 (9)	0.0152 (9)
C14	0.0535 (13)	0.0541 (13)	0.0596 (13)	0.0060 (11)	0.0206 (11)	0.0218 (11)
C15	0.0518 (14)	0.0765 (16)	0.0712 (15)	0.0216 (12)	0.0114 (12)	0.0337 (13)
C16	0.0481 (13)	0.0781 (17)	0.0953 (18)	0.0214 (13)	0.0246 (13)	0.0393 (15)
C17	0.0583 (15)	0.0719 (16)	0.0874 (18)	−0.0091 (13)	0.0331 (14)	0.0201 (14)
C18	0.0346 (10)	0.0358 (10)	0.0381 (10)	0.0098 (8)	0.0166 (8)	0.0135 (8)
C19	0.0347 (10)	0.0563 (12)	0.0515 (12)	0.0145 (9)	0.0175 (9)	0.0281 (10)
C20	0.0375 (11)	0.0590 (13)	0.0595 (12)	0.0122 (10)	0.0259 (10)	0.0311 (11)
C21	0.0425 (11)	0.0449 (11)	0.0464 (11)	0.0148 (9)	0.0238 (9)	0.0235 (9)
C22	0.0347 (10)	0.0528 (12)	0.0494 (11)	0.0139 (9)	0.0173 (9)	0.0249 (10)
C23	0.0355 (10)	0.0475 (11)	0.0528 (11)	0.0118 (9)	0.0230 (9)	0.0247 (9)
C24	0.0457 (12)	0.0632 (13)	0.0572 (12)	0.0085 (10)	0.0265 (10)	0.0281 (11)
C25	0.0545 (13)	0.0630 (14)	0.0597 (13)	0.0095 (11)	0.0300 (11)	0.0316 (12)
C26	0.0772 (17)	0.0818 (17)	0.0590 (15)	0.0070 (14)	0.0231 (13)	0.0290 (13)
C27	0.124 (3)	0.133 (3)	0.0605 (16)	0.028 (2)	0.0300 (17)	0.0471 (18)
O1	0.0568 (9)	0.0804 (11)	0.0435 (8)	0.0159 (8)	0.0266 (7)	0.0101 (7)
O2	0.0475 (9)	0.0647 (9)	0.0419 (8)	0.0189 (8)	0.0187 (7)	0.0051 (7)
O3	0.0485 (8)	0.0567 (9)	0.0406 (7)	0.0150 (7)	0.0227 (6)	0.0131 (6)
O4	0.0600 (10)	0.0686 (10)	0.0498 (9)	0.0072 (8)	0.0345 (8)	0.0070 (8)
O5	0.0462 (8)	0.0777 (10)	0.0729 (10)	0.0231 (7)	0.0334 (7)	0.0513 (8)

### 2,2'-[(4-Butoxyphenyl)methylene]bis(3-hydroxy-5,5-dimethylcyclohex-2-en-1-one) Geometric parameters (Å, º)

**Table:**

C1—O2	1.299 (2)	C15—H15A	0.9600
C1—C6	1.380 (2)	C15—H15B	0.9600
C1—C2	1.498 (3)	C15—H15C	0.9600
C2—C3	1.518 (3)	C16—H16A	0.9600
C2—H2A	0.9700	C16—H16B	0.9600
C2—H2B	0.9700	C16—H16C	0.9600
C3—C4	1.522 (3)	C17—H17A	0.9600
C3—C15	1.525 (3)	C17—H17B	0.9600
C3—C14	1.532 (3)	C17—H17C	0.9600
C4—C5	1.500 (3)	C18—C19	1.382 (2)
C4—H4A	0.9700	C18—C23	1.387 (2)
C4—H4B	0.9700	C19—C20	1.386 (2)
C5—O1	1.270 (2)	C19—H19	0.9300
C5—C6	1.419 (2)	C20—C21	1.381 (3)
C6—C7	1.526 (2)	C20—H20	0.9300
C7—C8	1.521 (2)	C21—O5	1.368 (2)
C7—C18	1.533 (2)	C21—C22	1.382 (2)
C7—H7	0.9800	C22—C23	1.372 (2)
C8—C13	1.374 (2)	C22—H22	0.9300
C8—C9	1.421 (2)	C23—H23	0.9300
C9—O3	1.268 (2)	C24—O5	1.424 (2)
C9—C10	1.494 (3)	C24—C25	1.504 (3)
C10—C11	1.528 (3)	C24—H24A	0.9700
C10—H10A	0.9700	C24—H24B	0.9700
C10—H10B	0.9700	C25—C26	1.507 (3)
C11—C12	1.524 (3)	C25—H25A	0.9700
C11—C17	1.528 (3)	C25—H25B	0.9700
C11—C16	1.533 (3)	C26—C27	1.491 (3)
C12—C13	1.494 (3)	C26—H26A	0.9700
C12—H12A	0.9700	C26—H26B	0.9700
C12—H12B	0.9700	C27—H27A	0.9600
C13—O4	1.314 (2)	C27—H27B	0.9600
C14—H14A	0.9600	C27—H27C	0.9600
C14—H14B	0.9600	O2—H2	0.927 (17)
C14—H14C	0.9600	O4—H4	0.942 (18)
			
O2—C1—C6	124.27 (17)	H14B—C14—H14C	109.5
O2—C1—C2	113.58 (16)	C3—C15—H15A	109.5
C6—C1—C2	122.16 (16)	C3—C15—H15B	109.5
C1—C2—C3	115.21 (16)	H15A—C15—H15B	109.5
C1—C2—H2A	108.5	C3—C15—H15C	109.5
C3—C2—H2A	108.5	H15A—C15—H15C	109.5
C1—C2—H2B	108.5	H15B—C15—H15C	109.5
C3—C2—H2B	108.5	C11—C16—H16A	109.5
H2A—C2—H2B	107.5	C11—C16—H16B	109.5
C2—C3—C4	107.34 (16)	H16A—C16—H16B	109.5
C2—C3—C15	110.27 (17)	C11—C16—H16C	109.5
C4—C3—C15	110.01 (17)	H16A—C16—H16C	109.5
C2—C3—C14	110.52 (17)	H16B—C16—H16C	109.5
C4—C3—C14	110.38 (17)	C11—C17—H17A	109.5
C15—C3—C14	108.32 (17)	C11—C17—H17B	109.5
C5—C4—C3	114.71 (15)	H17A—C17—H17B	109.5
C5—C4—H4A	108.6	C11—C17—H17C	109.5
C3—C4—H4A	108.6	H17A—C17—H17C	109.5
C5—C4—H4B	108.6	H17B—C17—H17C	109.5
C3—C4—H4B	108.6	C19—C18—C23	116.65 (16)
H4A—C4—H4B	107.6	C19—C18—C7	122.81 (15)
O1—C5—C6	122.08 (17)	C23—C18—C7	120.03 (15)
O1—C5—C4	116.78 (16)	C18—C19—C20	122.14 (17)
C6—C5—C4	121.10 (17)	C18—C19—H19	118.9
C1—C6—C5	117.87 (16)	C20—C19—H19	118.9
C1—C6—C7	123.85 (15)	C21—C20—C19	119.76 (17)
C5—C6—C7	118.23 (15)	C21—C20—H20	120.1
C8—C7—C6	114.91 (14)	C19—C20—H20	120.1
C8—C7—C18	113.69 (14)	O5—C21—C20	124.82 (16)
C6—C7—C18	115.36 (14)	O5—C21—C22	116.12 (16)
C8—C7—H7	103.6	C20—C21—C22	119.05 (17)
C6—C7—H7	103.6	C23—C22—C21	120.18 (17)
C18—C7—H7	103.6	C23—C22—H22	119.9
C13—C8—C9	117.57 (17)	C21—C22—H22	119.9
C13—C8—C7	120.39 (16)	C22—C23—C18	122.21 (17)
C9—C8—C7	121.95 (15)	C22—C23—H23	118.9
O3—C9—C8	121.85 (17)	C18—C23—H23	118.9
O3—C9—C10	116.66 (16)	O5—C24—C25	107.97 (16)
C8—C9—C10	121.46 (16)	O5—C24—H24A	110.1
C9—C10—C11	115.37 (16)	C25—C24—H24A	110.1
C9—C10—H10A	108.4	O5—C24—H24B	110.1
C11—C10—H10A	108.4	C25—C24—H24B	110.1
C9—C10—H10B	108.4	H24A—C24—H24B	108.4
C11—C10—H10B	108.4	C24—C25—C26	113.79 (19)
H10A—C10—H10B	107.5	C24—C25—H25A	108.8
C12—C11—C10	107.03 (16)	C26—C25—H25A	108.8
C12—C11—C17	109.85 (17)	C24—C25—H25B	108.8
C10—C11—C17	109.92 (18)	C26—C25—H25B	108.8
C12—C11—C16	110.96 (18)	H25A—C25—H25B	107.7
C10—C11—C16	110.65 (18)	C27—C26—C25	113.9 (2)
C17—C11—C16	108.43 (18)	C27—C26—H26A	108.8
C13—C12—C11	114.12 (16)	C25—C26—H26A	108.8
C13—C12—H12A	108.7	C27—C26—H26B	108.8
C11—C12—H12A	108.7	C25—C26—H26B	108.8
C13—C12—H12B	108.7	H26A—C26—H26B	107.7
C11—C12—H12B	108.7	C26—C27—H27A	109.5
H12A—C12—H12B	107.6	C26—C27—H27B	109.5
O4—C13—C8	123.47 (17)	H27A—C27—H27B	109.5
O4—C13—C12	114.02 (16)	C26—C27—H27C	109.5
C8—C13—C12	122.49 (17)	H27A—C27—H27C	109.5
C3—C14—H14A	109.5	H27B—C27—H27C	109.5
C3—C14—H14B	109.5	C1—O2—H2	113.2 (18)
H14A—C14—H14B	109.5	C13—O4—H4	113 (2)
C3—C14—H14C	109.5	C21—O5—C24	117.78 (14)
H14A—C14—H14C	109.5		
			
O2—C1—C2—C3	160.74 (17)	C9—C10—C11—C12	−46.3 (2)
C6—C1—C2—C3	−19.2 (3)	C9—C10—C11—C17	−165.60 (18)
C1—C2—C3—C4	47.6 (2)	C9—C10—C11—C16	74.7 (2)
C1—C2—C3—C15	167.44 (18)	C10—C11—C12—C13	50.3 (2)
C1—C2—C3—C14	−72.8 (2)	C17—C11—C12—C13	169.64 (18)
C2—C3—C4—C5	−48.9 (2)	C16—C11—C12—C13	−70.5 (2)
C15—C3—C4—C5	−168.94 (18)	C9—C8—C13—O4	170.37 (17)
C14—C3—C4—C5	71.6 (2)	C7—C8—C13—O4	−6.4 (3)
C3—C4—C5—O1	−159.53 (17)	C9—C8—C13—C12	−8.0 (3)
C3—C4—C5—C6	22.4 (3)	C7—C8—C13—C12	175.28 (17)
O2—C1—C6—C5	168.64 (17)	C11—C12—C13—O4	156.05 (18)
C2—C1—C6—C5	−11.4 (3)	C11—C12—C13—C8	−25.5 (3)
O2—C1—C6—C7	−8.7 (3)	C8—C7—C18—C19	−152.88 (17)
C2—C1—C6—C7	171.27 (17)	C6—C7—C18—C19	−17.1 (2)
O1—C5—C6—C1	−168.28 (18)	C8—C7—C18—C23	35.6 (2)
C4—C5—C6—C1	9.7 (3)	C6—C7—C18—C23	171.40 (16)
O1—C5—C6—C7	9.2 (3)	C23—C18—C19—C20	−0.6 (3)
C4—C5—C6—C7	−172.81 (17)	C7—C18—C19—C20	−172.40 (17)
C1—C6—C7—C8	83.6 (2)	C18—C19—C20—C21	1.1 (3)
C5—C6—C7—C8	−93.71 (19)	C19—C20—C21—O5	178.59 (17)
C1—C6—C7—C18	−51.6 (2)	C19—C20—C21—C22	−0.9 (3)
C5—C6—C7—C18	131.04 (17)	O5—C21—C22—C23	−179.26 (16)
C6—C7—C8—C13	90.6 (2)	C20—C21—C22—C23	0.3 (3)
C18—C7—C8—C13	−133.42 (17)	C21—C22—C23—C18	0.2 (3)
C6—C7—C8—C9	−86.00 (19)	C19—C18—C23—C22	0.0 (3)
C18—C7—C8—C9	50.0 (2)	C7—C18—C23—C22	171.99 (16)
C13—C8—C9—O3	−165.22 (17)	O5—C24—C25—C26	−67.1 (2)
C7—C8—C9—O3	11.5 (3)	C24—C25—C26—C27	−174.0 (2)
C13—C8—C9—C10	12.5 (3)	C20—C21—O5—C24	5.5 (3)
C7—C8—C9—C10	−170.83 (16)	C22—C21—O5—C24	−174.98 (17)
O3—C9—C10—C11	−165.42 (16)	C25—C24—O5—C21	174.82 (16)
C8—C9—C10—C11	16.8 (3)		

### 2,2'-[(4-Butoxyphenyl)methylene]bis(3-hydroxy-5,5-dimethylcyclohex-2-en-1-one) Hydrogen-bond geometry (Å, º)

*Cg*1 is the centroid of the C18–C22 benzene ring.

**Table:**

*D*—H···*A*	*D*—H	H···*A*	*D*···*A*	*D*—H···*A*
O2—H2···O3	0.92 (2)	1.66 (2)	2.566 (2)	166 (3)
O4—H4···O1	0.94 (3)	1.72 (3)	2.655 (2)	171 (3)
C10—H10*B*···*Cg*1^i^	0.97	3.11	3.773 (2)	127

Symmetry code: (i) −*x*+1, −*y*+1, −*z*+1.
